# Distinct doping dependence of critical temperature and critical current density in Ba_1−*x*_K_*x*_Fe_2_As_2_ superconductor

**DOI:** 10.1038/srep26671

**Published:** 2016-05-25

**Authors:** Dongjoon Song, Shigeyuki Ishida, Akira Iyo, Masamichi Nakajima, Jun-ichi Shimoyama, Michael Eisterer, Hiroshi Eisaki

**Affiliations:** 1 Electronics and Photonics Research Institute, National Institute of Advanced Industrial Science and Technology (AIST), Tsukuba 305-8568, Japan; 2 Department of Physics, Osaka University, Toyonaka, Osaka 560-0043, Japan; 3 Department of Physics and Mathematics, Aoyama Gakuin University, Sagamihara 252-5258, Japan; 4 Atominstitut, Vienna University of Technology, Stadionallee 2, 1020 Vienna, Austria

## Abstract

Since the high transition temperature (High-*T*_*c*_) superconductivity was discovered in the series of materials containing iron (Fe), their potential for the applications has been extensively scrutinized. In particular, a lot of effort has been made in achieving the high current-carrying ability by revealing the vortex pinning behavior. Here, we report on the critical current density (*J*_*c*_) for the pristine Ba_1−*x*_K_*x*_Fe_2_As_2_ single crystals with various K concentrations (0.25 ≤ *x* ≤ 0.52) determined by the magnetization hysteresis loop measurements. The *x*-dependence of *J*_c_ is characterized by a spike-like peak at *x* ~ 0.30, which corresponds to the under-doped region. This behavior is distinct from a moderate *T*_c_ dome with a broad maximum spanning from *x* ~ 0.3 to 0.5. For the under-doped samples, with increasing magnetic field (*H*), a second magnetization peak in *J*_c_ is observed, whereas for the optimally- and over-doped samples, *J*_c_ monotonically decreases with *H*. This result emphasizes that fine tuning of doping composition is important to obtain strong flux pinning. The origin of the characteristic doping dependence of *J*_*c*_ is discussed in connection with the orthorhombic phase domain boundary, as well as the chemical inhomogeneity introduced by the dopant substitutions.

A high superconducting transition temperature (*T*_c_), upper critical field (*H*_c2_), and critical current density (*J*_c_) are the three major requirements for large current and/or high magnetic field applications of superconductivity. Iron (Fe)-based superconductors discovered in 2008^1^ are considered to satisfy these requirements because of their relatively high *T*_c_ reaching 56 K at highest^2^, as well as their high *H*_c2_ exceeding 100 T^3^,^4^. In particular, materials derived from *AE*Fe_2_As_2_ (*AE* = alkaline earth elements), so-called 122 – type materials, were regarded as the most promising candidates, since they possess further attractive properties, such as the small anisotropy factor (*H*_*c2*_^//*ab*^/H_*c2*_^*//c*^ = γ  = 1~2)^5^,^6^, superior inter-grain connectivity^7^,^8^, and easiness in the sample synthesis *etc*. Various thin-films and bulk wires have been fabricated (mostly by powder-in-tube (PIT) methods) using 122-based materials^9^,^10^. To date, *J*_c_ values reach up to 10^6^ A/cm^2^ for a BaFe_2_(As_1−x_P_x_)_2_ thin film (4 K, 9 T)^11^ and 10^5^ A/cm^2^ for a Ba_1−*x*_K_*x*_Fe_2_As_2_ (or Sr_1−*x*_K_*x*_Fe_2_As_2_) PIT wire (4.2 K, 10 T)^12^,^13^, respectively.

While *T*_c_ and *H*_c2_ are intrinsic material parameters and thus more or less determined by the microscopic superconducting mechanism, *J*_c_ is effectively determined by vortex pinning in single crystals or films, which is either of intrinsic or extrinsic origin. In general, the pinning force is not strong in homogeneous high-quality single crystals without defects, while introduction of artificial disorder results in enhancement of *J*_c_. From the same point of view, in the case of Fe-based superconductors, many efforts have been paid to develop suitable materials with defect structures which give rise to strong vortex pinning, as well as to understand the fundamental pinning mechanism. As for the former, the introduction of columnar defects by irradiation with high energy particles^14^,^15^,^16^ or self-assembling BaFeO_2_ nanorods^17^,^18^ has been proven to be effective in increasing *J*_c_. As for the latter, it is recognized that the Fe-based superconductors exhibit a second magnetization peak (SMP) in *J*_c_^19^,^20^,^21^,^22^,^23^,^24^,^25^, which is associated with a peak at the finite magnetic field (*H*) appearing in the magnetization hysteresis loop (MHL). Thus, the study of the SMP effect is of great interest, from both academic and technological points of view.

So far, systematic doping (*x*) dependent studies of the Ba(Fe_1−*x*_Co_*x*_)_2_As_2_ and BaFe_2_(As_1−*x*_P_*x*_)_2_ single crystals have been reported^26^,^27^,^28^,^29^. It has been shown that a doping dependence of SMP and *J*_*c*_ are observed and the *J*_c_ tends to be high at particular doping concentrations. As for the source of pinning, various mechanism have been proposed, including domain boundary^26^,^30^,^31^, compositional disorder^21^,^29^, *etc*., as shall be discussed later. Still, a consensus has not been reached so far. Similarly, Ba_1−*x*_K_*x*_Fe_2_As_2_, the highest *T*_*c*_ and *H*_*c2*_ material among the 122-type superconductors^32^,^33^, is also known to show SMP^20^,^22^. However, to the best of our knowledge, there has been no systematic study on the *x*-dependence of *J*_c_ in spite of its highest potential as a material for future applications. In this work, we report the systematic evolution of the vortex pinning behavior and *J*_c_ in the Ba_1−*x*_K_*x*_Fe_2_As_2_ single crystals ranging from under- (*x* = 0.25) to over-doped (*x* = 0.52) compositions. We established a detailed *x* - *J*_c_ phase diagram, which is characterized by a spike-like peak at the slightly under-doped composition around *x* ~ 0.30. The behavior contrasts with the moderate dome-like *x*-dependence of the *T*_c_ with a broad maximum at *x* ~ 0.3 to 0.5. High *J*_c_ is apparently related to existence of the SMP which disappears at optimal- and over-doping composition. Possible mechanisms to account for the origin of the enhanced *J*_c_ will be discussed.

## Results

**Figure 1:**
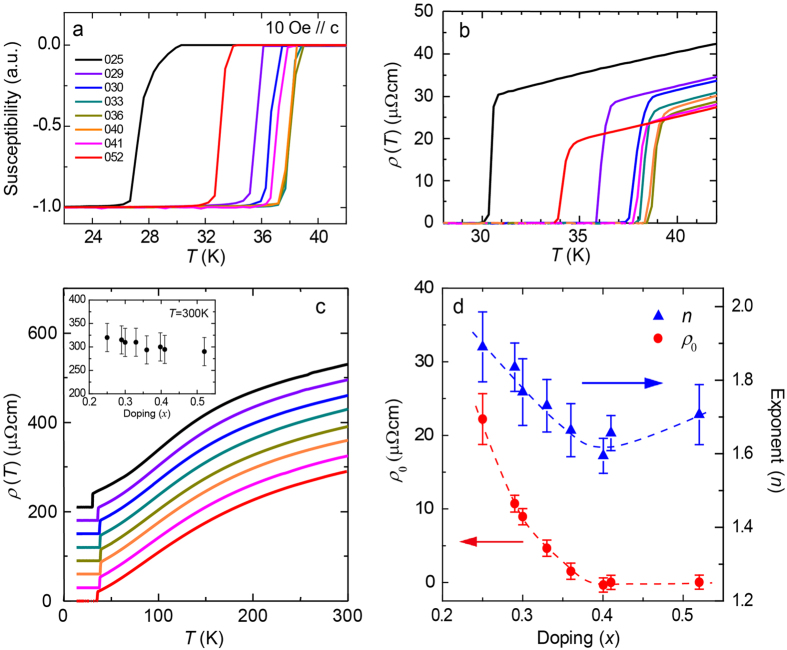
(**a**) Temperature dependence of the magnetic susceptibility of Ba_1−*x*_K_*x*_Fe_2_As_2_ (*x* = 0.25–0.52). (**b,c**) Temperature dependence of the in-plane resistivity, *ρ*(*T*), for the temperature range of 28 K to 42 K and 0 K to 300 K, respectively, with the same color cord used in (**a**). In (**c**), 30 μΩcm offset is applied for every doping increment and *x* dependence of room temperature resistivity, *ρ*(300 K), is shown in the inset. (**d**) Residual resistivity *ρ*_0_ and exponent *n* extracted from *ρ*(*T*) of the low temperature region by using a power-law fitting, *ρ*(*T*) = *ρ*_0_ + *AT *^*n*^.

Figure 1(a) shows the temperature (*T*)-dependence of the magnetic susceptibility (χ) for the Ba_1−*x*_K_*x*_Fe_2_As_2_ single crystal samples measured under zero-field-cooling (ZFC) conditions with *H* = 10 Oe applied along the *c*-axis. (For a better comparison, the data are normalized by the magnitude at 5 K). The superconducting transition is sharp with Δ*T*_c_ < 0.5 K except for *x* = 0.25, indicative of good sample quality. With increasing *x*, *T*_c_ increases from 27.5 K for *x* = 0.25 to 38.5 K for *x* = 0.36, then gradually decreases with further doping, down to 33 K for *x* = 0.52. We also performed the in-plane resistivity (*ρ*) measurements on these samples. The results are plotted in Fig. 1(b) (28 K ≤ *T* ≤ 42 K) and (c) (0 K ≤ *T* ≤ 300 K), respectively. We used the same color code as in Fig. 1(a). In Fig. 1(b), the superconducting transition of Ba_1−*x*_K_*x*_Fe_2_As_2_ is very sharp (*ΔT*_c_ < 0.5 K) and shows a *x*-dependence. *T*_c_’s defined as the zero resistance *T*^ ′^s are in good agreement with those defined by *χ*, which exhibits a dome-like shape with the broad maximum spanning from *x* ~ 0.3 to 0.5.

In Fig. 1(c), each *ρ* curve is shifted by 30 μΩcm to avoid an overlap. In the inset, the absolute value of *ρ* at 300 K, *ρ*(300 K), is plotted, which shows gradual decrease from 320 μΩcm (*x* = 0.25) to 290 μΩcm (*x* = 0.52). The overall shape of *ρ*(*T*) is similar with each other, which shows the saturating behavior at the high *T* region, in other words, S-shaped *T*-dependence. For *x* = 0.25, anomaly in *ρ* is observed at around 65 K due to the anti-ferromagnetic/orthorhombic phase transition. In the low-*T* region, *ρ*(*T*)s are well fitted by a power-law function, *ρ*(*T*) = *ρ*_0_ + *AT *^*n*^ (the details of fitting procedure are described in Method section). The estimated residual resistivity *ρ*_0_ and exponent *n* are shown in Fig. 1(d), which will be discussed later.

**Figure 2:**
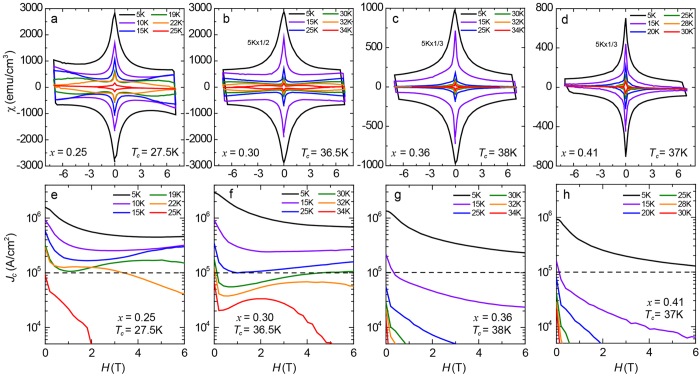
(**a–d**) Magnetization hysteresis loops (MHL) of Ba_1−*x*_K_*x*_Fe_2_As_2_ with *x* = 0.25, 0.30, 0.36, and 0.41 measured at several temperatures. (**e,f**) Magnetic field and temperature dependence of the critical current density *J*_c_ determined by the Bean model from the MHL shown in (**a–d**), respectively. Dashed horizontal lines indicate the practical-level of *J*_c_ = 10^5^ A/cm^2^.

Figure 2(a–d) show typical MHLs measured at various *T* with *H* along the *c* axis. Each sample represents under-doped (*x* = 0.25, *T*_c_ = 27.5 K), slightly under-doped (*x* = 0.30, *T*_c_ = 36.5 K), optimally doped (*x* = 0.36, *T*_c_ = 38 K), and slightly over-doped (*x* = 0.41, *T*_c_ = 37 K) concentration, respectively. Qualitatively, the overall features measured at *T* = 5 K are more or less similar to each other in that they are characterized by a sharp peak centered at *H* = 0 and almost symmetric shapes with respect to *H*. The latter property indicates the dominant contribution of bulk pinning instead of a surface barrier^34^. These behaviors are also seen in various Fe-based superconductors. The width of the irreversible magnetization Δ*M* tends to shrink with increasing *x*. Moreover, at higher temperatures (*T* > 15 K), for *x* = 0.25 and 0.30, *M* increases with *H* after the initial decrease in the low *H* region, indicative of the SMP. The broad peaks are evident above 19 K for *x* = 0.25 and 30 K for *x* = 0.30, respectively. The position of the SMP moves towards higher *H* as *T* decreases and eventually goes beyond the accessible field range, *H* = 7 T in the present case. In contrast, for *x* = 0.36 and 0.41, *M* decreases monotonically with *H* at all *T*.

To determine *J*_c_ from the MHLs, we employed the extended Bean model^35^, *J*_c_ = 20▵*M*/[*w*(1 − *w*/3*l*)], where *w* and *l* are the dimensions of the rectangular sample (*w* < *l*). Figure 2(e–h) show the *H*-dependent *J*_c_ corresponding to the compositions shown in Fig. 2(a–d). The dashed lines indicate *J*_*c*_ = 10^5^ A/cm^2^, which is a threshold value for practical applications. At *T* = 5 K, *J*_c_ is the order of 10^6^ A/cm^2^ at zero field, 1.6 × 10^6^ A/cm^2^ (*x* = 0.25), 3.0 × 10^6^ A/cm^2^ (*x* = 0.30), 1.4 × 10^6^ A/cm^2^ (*x* = 0.36), and 0.9 × 10^6^ A/cm^2^ (*x* = 0.41), respectively. Among the four samples, *J*_c_ is the highest at *x* = 0.30. This crystal is slightly under-doped in terms of *T*_c_, since *T*_c_ of the *x* = 0.30 sample is 36.5 K, lower than the highest *T*_c_ of 38 K at *x* = 0.36. The result shows that the highest *J*_*c*_ composition does not match the highest *T*_*c*_ composition in Ba_1−*x*_K_*x*_Fe_2_As_2_. With increasing *H*, *J*_c_ decreases monotonically, while keeping high values above 10^5^ A/cm^2^ up to *H* = 6 T, 4.6 × 10^5^ A/cm^2^ (*x* = 0.25), 6.8 × 10^5^ A/cm^2^ (*x* = 0.30), 2.3 × 10^5^ A/cm^2^ (*x* = 0.36), and 1.3 × 10^5^ A/cm^2^ (*x* = 0.41), respectively, indicating the high current-carrying ability of this system at low *T*.

At higher *T*, *J*_c_ of the *x* = 0.25 and 0.30 samples exhibit a non-monotonic *H* dependence reflecting the SMP effect. As a consequence, the *x* = 0.30 sample sustains *J*_c_ exceeding 10^5^ A/cm^2^ even at *T* = 25 K and *H* = 6 T, showing the possibility of high magnetic field applications with an operation temperature above 20 K even without introducing artificial pinning center. On the other hand, the optimal- (*x* = 0.36) and over-doped (*x* = 0.41) samples always exhibit a monotonic decrease of *J*_c_ with *H*, resulting in a much lower *J*_c_ under high *H*.

**Figure 3:**
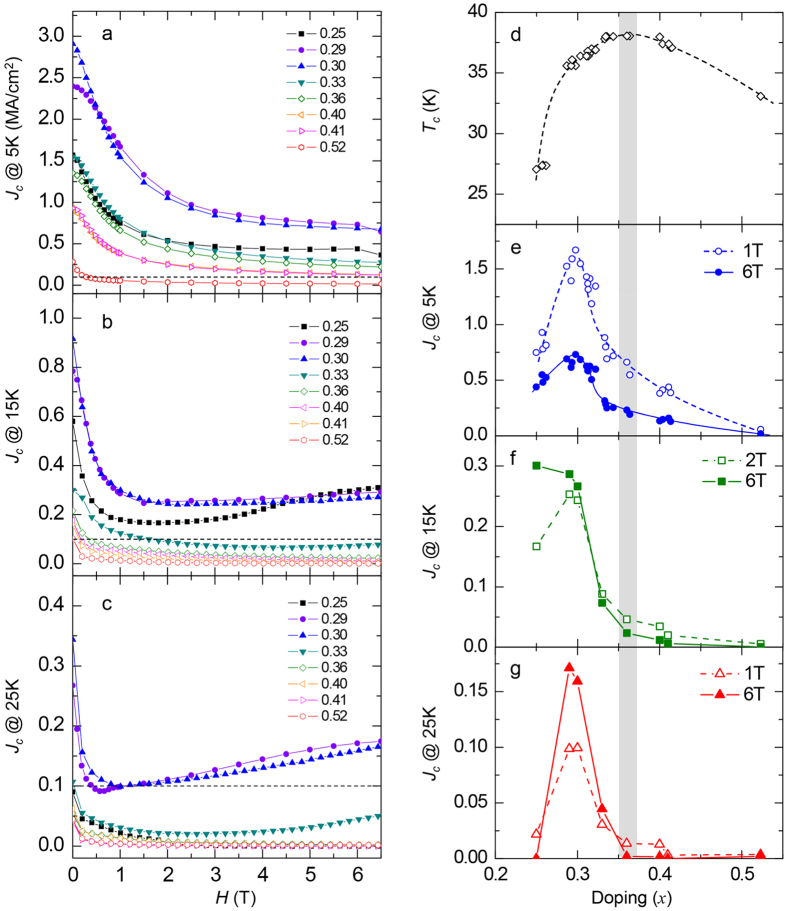
(**a–c**) Doping dependence of *J*_c_ as a function of magnetic field at 5 K, 15 K, and 25 K for *x* = 0.25, 0.29, 0.30, 0.33, 0.36, 0.40, 0.41, and 0.52. (**d**) Doping dependence of *T*_c_ for 26 samples. (**e**) *J*_*c*_ of the 26 samples at *T* = 5 K, *H* = 1 and 6 T. (**f,g**) Doping dependence of *J*_c_ at *T* = 15 and 25 K, respectively, with *H* = 1, 2 and 6 T extracted from (**b,c**). The shaded region in panels (**d–g**) indicates the optimal doping region, *x* ~ 0.36, showing maximum *T*_c_ ~ 38.5 K.

For a more detailed comparison, *H*-dependence of *J*_*c*_ for various *x*’s (*x* = 0.25, 0.29, 0.30, 0.33, 0.36, 0.40, 0.41, 0.52) at 5 K, 15 K and 25 K are plotted in Fig. 3(a–c). For each *T*, *J*_c_ changes more than tenfold with changing *x*. At *T* = 5 K (Fig. 3(a)), crystals with *x* = 0.29 and 0.30 possess the highest *J*_*c*_ among all compositions. On the other hand, *J*_c_ rapidly decreases either with increasing or decreasing *x*. The resultant *x*-dependence is shown in Fig. 3(e), in which the *J*_c_ values at *H* = 1 and 6 T for 26 samples are plotted. A peak in *J*_*c*_ is observed around *x* = 0.30. The peak is sharp with its full width half maximum as small as 0.08. Therefore, the *J*_c_-*x* phase diagram turns out to be very different from the *T*_c_-*x* phase diagram which is shown in Fig. 3(d). First, the peak position is *x* = 0.30 for the former, while *x* = 0.36 for the latter. Second, *J*_c_ shows salient *x*-dependence, while *T*_c_ changes mildly with *x*.

At 15 K (Fig. 3(b)), non-monotonic *H*-dependence is seen for *x* = 0.25, *x* = 0.29, and *x* = 0.30. For these samples, *J*_c_ first decreases with *H*, then increases at high *H*. This behavior reflects the SMP observed in the MHL. In detail, the minimum *J*_c_ value of *x* = 0.25 at *H* = 1.8 T is smaller than that of *x* = 0.29 and 0.30 at *H* = 1.5 T and 2.2 T, respectively. On the contrary, the enhancement of *J*_c_ at high field is more prominent for *x* = 0.25 than that of *x* = 0.29 and 0.30, presumably because of the strong SMP effect of *x* = 0.25. Consequently, for the *H* = 6 T result shown in Fig. 3(f), the *x* = 0.25 sample exhibits the highest *J*_c_, which is due to the steep increase of *J*_c_ with field. Meanwhile, as *x* increases above 0.33, *J*_c_ at *H* = 0 becomes smaller with *x* and decreases monotonically with *H* (Fig. 3(b)). As a result, *J*_c_ remains low (typically below 10^5^ A/cm^2^) in the entire *H*-region.

At 25 K (Fig. 3(c)), with disappearance of the SMP, *J*_c_ becomes significantly lower for *x* = 0.25 compared to *x* = 0.29 and *x* = 0.30. This is because the measurement *T* is close to *T*_c_ of the *x* = 0.25 sample (*T*_*c*_ = 27.5 K). For *x* = 0.29 and 0.30, non-monotonic *H*-dependence persists and *J*_c_’s above 10^5^ A/cm^2^ are recorded up to *H* = 6 T except for the small *H* range around 0.5 T and 1 T, respectively. On the other hand, for *x* = 0.33 and above, *J*_c_ decreases down to the 10^4^ A/cm^2^ range, although they possess *T*_c_ much higher than the measurement *T*.

## Discussion

As shown in Fig. 3(d–g), *J*_c_ and *T*_c_ of the Ba_1−*x*_K_*x*_Fe_2_As_2_ single crystal samples show contrasting *x*-dependence. A distinct *x*-dependence of *J*_c_ and *T*_c_ is also reported for the Ba(Fe_1−*x*_Co_*x*_)_2_As_2_ and BaFe_2_(As_1−x_P_x_)_2_ single crystals^26^,^27^,^28^,^29^. In both cases, *J*_*c*_ exhibits marked *x*-dependence with a peak near the left end of the superconducting dome (under-doped region), while *T*_*c*_ gradually changes with *x*. Based on the Ba(Fe_1−*x*_Co_*x*_)_2_As_2_ results, R. Prozorov *et al*.^26^ ascribed the characteristic *J*_*c*_ behavior to intrinsic pinning on the structural domains in the orthorhombic phase, which neighbors the superconducting phase on the under-doped side. On the other hand, based on the BaFe_2_(As_1−*x*_P_*x*_)_2_ single crystal results, S. Demirdiş *et al*.^29^ and L. Fang *et al*.^28^ proposed that the inhomogeneity in dopant distribution causes a spatial fluctuation of the superconducting condensation energy (δ*T*_c_ pinning) and/or the mean free path (δ*l* pinning)^36^. To our knowledge, most of the precedent MHL studies take the latter stance.

Existing phase diagrams of the Ba_1−*x*_K_*x*_Fe_2_As_2_ system^37^,^38^ show that the orthorhombic phase disappears between *x* = 0.25 and 0.30, which approximately matches with the composition where the enhancement of *J*_c_ is found. In this regard, the present results are compatible with the idea that the orthorhombic structural domains are the main source of vortex pinning. On the other hand, comparing Fig. 3(d,e), one notices that the high *J*_c_ is realized where the *x*-dependence of *T*_c_ (defined by *dT*_c_/*dx*) is large. In such a situation, small compositional inhomogeneity results in large local variation in *T*_c_, yielding δ*T*_c_ pinning. In addition, the possible role of δ*l* pinning to the enhanced *J*_c_ is suggested from the *x*-dependence of the residual resistivity *ρ*_0_. In Fig. 1(d), exponent *n* decreases gradually with *x* from ~1.9 at *x* = 0.25 to ~1.6 at *x* = 0.40, then slightly increases to ~1.7 at *x* = 0.52. This result indicates that the *T*-linear contribution becomes large at around optimal *x*. However, the feature is not significant compared to BaFe_2_(As_1−*x*_P_*x*_)_2_ case^39^ which shows certain variation in *n* from 2 to 1. On the other hand, *ρ*_0_ rapidly decreases by more than twenty times from *x* = 0.25 to *x* = 0.40. In this doping range, carrier density **is considered to evolve moderately, since the small change in the magnitude of *ρ*(300 K) as well as the small variation of *n*, ~1.6 < *n* < ~1.9, are observed. (see inset of Fig. 1(c)) Thus, it is natural to assume that the change in *ρ*_0_ comes from the change in the mean free path (*l*) of the carriers, not due to the change in the carrier number^40^. From the same point of view, the *x*-dependence of *ρ*_0_ in Fig. 1(d) reflects the *x*-dependence of *l*, which indicates that the δ*l* pinning should be stronger in the under-doped samples, while it becomes weaker in the optimal- to over-doped samples. This tendency consistently explains the observed *J*_c_ behavior. Meanwhile, we should not rule out another possibility that some novel features such as quantum criticality and/or anti-ferromagnetic/orbital fluctuations are correlated with the origin of strong flux pinning of this system.

In any case, the present results clearly demonstrate that intra-grain *J*_*c*_ of the Ba_1−*x*_K_*x*_Fe_2_As_2_ system takes the largest value at *x* = 0.30, which is different from the composition *x* = 0.40, commonly employed for fabricating PIT wires^12^. Although the real wires are composed of polycrystalline samples and their *J*_*c*_’s are affected by the inter-grain connectivity, as well as by the presence of pinning centers introduced either intentionally or accidentally, the present results suggest that *J*_*c*_ of PIT wires can be further increased by tuning the composition towards the lower doping side. This highlights the importance of fine chemical tuning for establishing and broadening the application potential of the Ba_1−*x*_K_*x*_Fe_2_As_2_ superconductor.

## Methods

The Ba_1−*x*_K_*x*_Fe_2_As_2_ single crystals with doping concentration *x* = 0.25–0.52 were grown by the KAs self-flux method using a stainless-steel container, following the method depicted in ref. 41. The doping concentration of the grown crystals was successfully controlled by tuning the mixing ratio of Ba and Fe in the starting compositions. In total, ten batches of Ba_1−*x*_K_*x*_Fe_2_As_2_ single crystals (two batches for *x* ~ 0.25, two batches for *x* ~ 0.3, two batches for *x* ~ 0.33, one batch for *x* ~ 0.36, two batches for *x* ~ 0.4, and one batch for *x* ~ 0.5, respectively) were grown for this study in order to check the reproducibility carefully. The compositions of the single crystals were confirmed by energy-dispersive X-ray (EDX) analysis and X-ray diffraction using Cu Kα radiation. The *c*-axis lengths determined by the X-ray diffraction were consistent with the compositions determined by EDX. The samples were cut into rectangular shapes with typical dimensions of 1 mm (length) × 0.8 mm (width) × 0.02 mm (thickness) for the magnetization and resistivity measurements. The *T*- and *H*- dependence of the magnetization were measured using a magnetic property measurement system (MPMS, Quantum Design). The resistivity measurements were carried out by a standard four probe method using a physical property measurement system (PPMS, Quantum Design). The data are reproducible and independent of the sample batch. We therefore believe that the present results represent intrinsic property of Ba_1−*x*_K_*x*_Fe_2_As_2_ system. We fitted the *ρ*(*T*) data using a power law function, *ρ*(*T*) = *ρ*_0_ + *AT *^*n*^, in the *T* range, *T*_*c*_ *<* *T* *<* *T*_*up*_. The upper bound temperature (*T*_*up*_) of fitting for each sample is taken well below the inflection point of S-shaped *ρ*(*T*) in order to avoid underestimation of exponent *n*. Since the inflection point shifts from *T* ~ 95 K to *T* ~ 80 K as *x* increases from *x* = 0.25 to *x* = 0.52^42^, *T*_*up*_ also gradually decreases from 80 K to 53 K.

## Additional Information

**How to cite this article**: Song, D. *et al*. Distinct doping dependence of critical temperature and critical current density in Ba_1−*x*_K_*x*_Fe_2_As_2_ superconductor. *Sci. Rep*. **6**, 26671; doi: 10.1038/srep26671 (2016).
